# An Advanced Pharmacy Practice Framework for Australia

**DOI:** 10.3390/pharmacy3020013

**Published:** 2015-06-09

**Authors:** Shane Jackson, Grant Martin, Jennifer Bergin, Bronwyn Clark, Ieva Stupans, Gilbert Yeates, Lisa Nissen, Stephen Marty, Paul Gysslink, Andrew Matthews, Sue Kirsa, Kerry Deans, Kay Sorimachi

**Affiliations:** 1 Advanced Pharmacy Practice Framework Steering Committee, P.O. Box 42, Deakin West, ACT 2600, Australia; E-Mails: kerry.deans@netspeed.com.au (K.D.); kay.sorimachi@psa.org.au (K.S.); 2 Australian Association of Consultant Pharmacy, P.O. Box 7071, Canberra Business Centre, ACT 2610, Australia; E-Mail: grant.martin@aacp.com.au; 3 Australian College of Pharmacy, P.O. Box 493, Fyshwick, ACT 2609, Australia; E-Mail: jenny.bergin@acp.edu.au; 4 Australian Pharmacy Council, P.O. Box 269, Civic Square, ACT 2608, Australia; E-Mail: bronwyn.clark@pharmacycouncil.org.au; 5 Council of Pharmacy Schools: Australia and New Zealand Inc., Faculty of Pharmacy, The University of Sydney, NSW 2006, Australia; E-Mail: ieva.stupans@une.edu.au; 6 Pharmaceutical Defence Limited, 40 Burwood Road, Hawthorn, VIC 3122, Australia; E-Mail: gilbert.yeates@pdlappco.com.au; 7 Pharmaceutical Society of Australia, P.O. Box 42, Deakin West, ACT 2600, Australia; E-Mail: l.nissen@qut.edu.au; 8 Pharmacy Board of Australia, G.P.O. Box 9958, Melbourne, VIC 3001, Australia; E-Mail: stephen.marty@ahpra.gov.au; 9 Professional Pharmacists Australia, G.P.O. Box 1272, Melbourne, VIC 3001, Australia; E-Mail: pgysslink@vtown.com.au; 10 The Pharmacy Guild of Australia, P.O. Box 7036, Canberra Business Centre, ACT 2610, Australia; E-Mail: andrew.matthews@pharmacycouncil.org.au; 11 The Society of Hospital Pharmacists of Australia, P.O. Box 1774, Collingwood, VIC 3066, Australia; E-Mail: sue.kirsa@petermac.org

**Keywords:** advanced practice, pharmacy, competency, standards, framework

## Abstract

The need to develop *An Advanced Pharmacy Practice Framework for Australia* (the “APPF”) was identified during the 2010 review of the competency standards for Australian pharmacists. The Advanced Pharmacy Practice Framework Steering Committee, a collaborative profession-wide committee comprised of representatives of ten pharmacy organisations, examined and adapted existing advanced practice frameworks, all of which were found to have been based on the *Competency Development and Evaluation Group (CoDEG) Advanced and Consultant Level Framework* (the “CoDEG Framework”) from the United Kingdom. Its competency standards were also found to align well with the Domains of the *National Competency Standards Framework for Pharmacists in Australia* (the “National Framework”). Adaptation of the CoDEG Framework created an APPF that is complementary to the National Framework, sufficiently flexible to customise for recognising advanced practice in any area of professional practice and has been approved by the boards/councils of all participating organisations. The primary purpose of the APPF is to assist the development of the profession to meet the changing health care needs of the community. However, it is also a valuable tool for assuring members of the public of the competence of an advanced practice pharmacist and the quality and safety of the services they deliver.

## 1. Introduction

The work to develop *An Advanced Pharmacy Practice Framework for Australia* [1] (the “APPF”) began in early 2011 under the auspices of the Advanced Pharmacy Practice Framework Steering Committee (APPFSC) as part of a collaborative profession-wide project. The APPFSC involved ten Australian pharmacy organisations (as listed under each author’s affiliation), which are considered to cover the broad interests of professional practice of pharmacists.

The desirability of undertaking work directed at gaining recognition for advanced pharmacy practice in Australia had been identified by the Competency Standards Review Steering Committee (CSRSC) during the 2010 review of the pharmacy profession’s competency standards. The outcome of that work was the release of the *National Competency Standards Framework for Pharmacists in Australia* (the “National Framework”) [2].

During the review preliminary work was undertaken to describe and define advanced practice. The adapted definition [3], which has been endorsed by participating pharmacy organisations is as follows:

*Advanced Practice is practice that is so significantly different from that achieved at initial registration that it warrants recognition by professional peers and the public of the expertise of the practitioner and the education, training and experience from which that capability was derived*.

Following the release of the National Framework, ongoing work around advanced pharmacy practice was considered vital to enhance the capabilities of individual pharmacists and the profession as a whole, but also to improve the profession’s capacity for responding to future changes in practice and the practice environment.

The APPFSC was established in March 2011 with the same profession-wide representation as the CSRSC. The decision to proceed with a similarly constituted group reflected the effectiveness of the working relationships that had been established in the group, but also provided for continuity in the expertise within the Committee for addressing competency-related issues on behalf of the profession. Additionally, ongoing broad coverage of the pharmacy sector was considered important for securing sector-wide support for the work and for assuring access to additional relevant professional expertise.

At its first meeting, the APPFSC noted the work already undertaken in the United Kingdom by the Competency Development and Evaluation Group (CoDEG) in describing an advanced practice framework for pharmacists [4]. The CoDEG is a collaborative network of pharmacists who, through research and evaluation, develop and publish frameworks covering different levels of practice (e.g., general level, advanced level) and practice areas. The frameworks and supporting tools made available by CoDEG are designed to help develop and support pharmacists and ensure their fitness to practise at all levels.

The APPFSC also noted that considerable progress had already been made in the hospital pharmacy sector in Australia to define advanced practice frameworks for a number of clinical care areas [5]. A review of each of these initiatives was considered to be appropriate initial work for the APPFSC to assess the usefulness and relevance of work already completed, and to determine the future work schedule for the Committee.

Release of the APPF to the profession occurred only after all boards and councils of the organisations represented on the APPFSC had approved the APPF and it had been endorsed by the Pharmacy Board of Australia. This document describes the work undertaken by the APPFSC to develop and gain the desired approvals and endorsement of the APPF.

## 2. Methods

The development of the APPF involved the following steps:

(1) examination of advanced level frameworks developed in Australia
(2) analysis of the CoDEG Advanced and Consultant Level Framework (the “CoDEG Framework”)
(3) mapping of the CoDEG Framework to the National Framework
(4) customisation of the CoDEG Framework for Australia
(5) development of the APPF for “patient care”
(6) testing adaptation of the APPF for “management and administration”
(7) preliminary approval of a draft APPF by supporting pharmacy organisations
(8) profession-wide consultation and modification of the draft APPF based on feedback
(9) approval of the final APPF by supporting pharmacy organisations
(10) endorsement by the Pharmacy Board of Australia
(11) release of the APPF to the profession

The APPFSC reviewed five examples of advanced level frameworks that were made available through the Society of Hospital Pharmacists of Australia. These frameworks, some of which were still work in progress, had been developed for use in Australia in defined clinical care areas encompassing paediatrics, critical care, emergency medicine, cardiology and oncology.

The CoDEG Framework was also used as a core reference in our analyses and subsequent decision-making. It is designed as a generic framework to guide the practitioner’s self-appraisal processes, work place assessment and career progression. There are six competency cluster areas in the CoDEG Framework: Expert Professional Practice; Building Working Relationships; Leadership; Management; Education, Training and Development; and Research and Evaluation. Each cluster area consists of several competencies (e.g., competencies on “communication” and “teamwork and consultation” are under the Building Working Relationships cluster), each of which is associated with competency level descriptors at three levels of practice. The APPFSC noted the relevance of the scope of the competencies included and the articulation of each competency at three different advanced performance levels as important features.

The content and structure of the APPF was analysed with particular reference to comparing its scope with the Domains of professional activity encompassed within the National Framework (refer to Table 1). The comparison underpinned decisions as to how the CoDEG Framework could be adapted to better suit the Australian practice environment. Since the existing frameworks developed in Australia all addressed areas of clinical practice, the initial adaptation and customisation of the CoDEG Framework was undertaken to create an advanced practice framework in “patient care”. The flexibility of the component parts of this framework for describing advanced practice in other areas of professional practice was subsequently tested using the example of “management and administration”.

**Table 1:** Domains of the National competency standards framework for pharmacists in Australia [2].

Domain Number	Competency Standard Area
Domain 1	Professional and ethical practice
Domain 2	Communication, collaboration and self-management
Domain 3	Leadership and management
Domain 4	Review and supply prescribed medicines
Domain 5	Prepare pharmaceutical products
Domain 6	Deliver primary and preventive health care
Domain 7	Promote and contribute to optimal use of medicines
Domain 8	Critical analysis, research and education

In late 2011, the APPFSC released a Discussion Paper to the boards and councils of the pharmacy organisations represented on the APPFSC and sought preliminary approval of the draft APPF. The Paper included contextual detail, definitions, a description of how the proposed framework had been developed, the advanced practice framework in “patient care” and demonstration of the customisation needed to create an advanced practice framework in “management and administration”. The boards and councils of APPFSC organisations also supported engaging the profession in a consultative process.

The Discussion Paper submitted to the governing bodies of participating organisations was revised in accordance with the feedback received to create a Consultation Paper. This was used to undertake profession-wide consultation for an eight-week period from March to May 2012. Responses were sought on a number of issues of interest to the APPFSC, and there was also scope for respondents to comment on any issue of concern. The responses from the profession were used to further refine the framework to create the final APPF. This was forwarded to the boards and councils of all participating pharmacy organisations for approval. The approved APPF was subsequently forwarded to the Pharmacy Board of Australia with a request for endorsement.

## 3. Results

### 3.1. Selecting an Appropriate Framework Structure

Rather than undertake a detailed analysis of the practice area-specific content, each of the sample Australian frameworks was reviewed to clarify the general approach used and the areas in which customisation had been undertaken. All five frameworks had referenced or adapted the CoDEG Framework with additional content included in the “Expert Professional Practice” cluster of competencies to show the specific knowledge and skills required in the clinical area of practice for which the framework had been or was being created. Thus, not surprisingly, the main differences in the Australian-adapted frameworks from the CoDEG Framework were evident in (although not limited to) the “Expert Professional Practice” area.

In addition to the relevance of scope and the articulation of three levels of advanced practice competencies, the potential value of the CoDEG Framework to the work of the APPFSC was significantly enhanced by the fact that Australian pharmacists had seen it as a relevant tool for adapting for their own use.

In the process of selecting a preferred framework structure for Australia, the APPFSC also considered work from other countries.

For example, work published by the Council on Credentialing in Pharmacy was insightful as it presented, among other things, connections between competency area, advanced level practice, scope of practice and credentialing in a clear manner [6].

### 3.2. Mapping the CODEG Framework to the Profession’s Competency Standards

Comparison of the CoDEG Framework with the National Framework revealed similarities in both structure and content, although no explicit link was apparent between the two Frameworks. By undertaking a mapping exercise it was possible to identify an overlap in the competency standards such that each of the CoDEG standards could be aligned to a Domain of the National Framework. This alignment was used to provide the desired continuum between the “general” and “advanced” level competency standards.

A high level summary of the result of the mapping between the CoDEG Framework and the National Framework is shown in Table 2 (with further detail available in Appendix 1 of the APPF [1]).

**Table 2:** Summary of comparison of the CoDEG Framework and the National Framework.

CoDEG Framework: Cluster	National Framework: Domain
1. Expert professional practice (includes Expert skills and knowledge, Patient care responsibilities, Reasoning and judgement, Professional autonomy)	4. Review and supply prescribed medicines 5. Prepare pharmaceutical products 6. Deliver primary and preventive care 7. Promote and contribute to optimal use of medicines
2. Building working relationships (includes Communication, Teamwork and consultation)	1. Professional and ethical practice 2. Communication, collaboration and self-management 8. Critical analysis, research and education
3. Leadership (includes Strategic content, Clinical governance, Vision, Innovation, Service development, Motivational)	3. Leadership and management
4. Management (includes Implementing national priorities, Resource utilisation, Standards of practice, Managing risk, Managing performance, Project management, Managing change, Strategic planning, Working across boundaries)	1. Professional and ethical practice 2. Communication, collaboration and self-management 3. Leadership and management 6. Deliver primary and preventive care 8. Critical analysis, research and education
5.Education, training and development (includes Role model, Mentorship, Conducting education and training, Continuing professional development, Links practice and education, Educational policy)	1. Professional and ethical practice 3. Leadership and management 8. Critical analysis, research and education
6. Research and evaluation (includes, Critical evaluation, Identifies gaps in the evidence base, Develops and evaluates research protocols, Creates evidence, Research evidence into practice, Supervises others undertaking research, Establishes research partnerships)	8. Critical analysis, research and education

### 3.3. Refining and Testing the Australian Advanced Level Framework

Similar to the CoDEG Framework, a three-tiered structure was retained in the Australian APPF to provide the capacity to differentiate advanced pharmacy practice across a continuum of performance levels.

The flexibility of the adapted CoDEG Framework was tested by using it to develop an APPF for “patient care”, and then customising that Framework for the area of practice of “management and administration”. The adapted framework could be used for any area of practice by customising those standards in the section labelled “Expert professional practice”. All other competency standards could be considered to have universal applicability to advanced pharmacy practice regardless of the area of practice and customisation was therefore considered unnecessary.

### 3.4. Defining Scope of Practice and Prerequisite Competencies

During the mapping exercise work was also undertaken to explore the use of the National Framework for describing the breadth of professional practice or “scope of practice”. In this exercise, the National Framework was used to describe the scope of practice of a pharmacist working in patient care at an “advanced” level and at a “general” level and it was found that the scope of practice for “advanced” level practice was significantly different to that which was generated for a pharmacist working at a “general” level. This is further discussed later in this paper.

A comparison of the two scopes of practice showed a linkage between the listed competencies and the expected performance level of that practitioner. Thus, these competencies were considered to be the prerequisite competencies for progression to working with the “advanced” level competencies. This resulted in the inclusion of components in the APPF, as shown in Table 3.

**Table 3:** Component parts of the APPF [1].

Section	Sub-part	Description
Part 1—“General” level requirements		Prerequisite competency standards drawn from the National Framework. All pharmacists seeking to achieve advanced level competence should first demonstrate they are able to meet these standards.
Part 2—General Career Characteristics		Guidance on general characteristics of pharmacists seeking to achieve advanced pharmacy practice. These characteristics are considered likely to be consistent for all areas of professional practice.
Part 3—“Advanced” level components	Part 3.1	Scope of practice for advanced pharmacy practice drawn from the National Framework. The process of defining scope of practice using the National Framework is described within that publication (refer to steps 1–4 on p. 6 of the National Framework) and can be replicated for other areas of practice for which an advanced practice framework is considered desirable.
	Part 3.2	Competency standards for advanced pharmacy practice which have been either reproduced or adapted from the CoDEG Framework with some changes to terminology considered desirable in the Australian context. It retains the three performance levels of the CoDEG Framework to provide a basis for pharmacists to self-assess their progress.

### 3.5. Profession-Wide Consultation

In mid-2011, approval was granted for the APPFSC to adapt the CoDEG Framework for use in this project. In October 2011, a Discussion Paper was disseminated to the boards and councils of participating organisations through their representatives on the APPFSC seeking their support for the draft APPF and for development of a Consultation Paper for dissemination within the profession. All organisations gave their approval for the proposed work and development of the APPF proceeded through the APPFSC. The boards and councils of participating organisations provided input (there were no substantial changes) to finalise the Consultation Paper, which was released to the profession in March 2012.

Respondents provided feedback on any or all of the twelve consultation issues which included the adequacy and appropriateness of key terms (e.g., scope of practice) and concepts (e.g., proposed component parts of the advanced practice framework, and the proposed three-level competency continuum), the linkages to the National Framework (e.g., to facilitate an understanding by pharmacists around practice progression and professional growth), and the development of a possible pathway to formally recognise advanced level pharmacists (e.g., possible credentialing criteria).

**Table 4:** Summary of competencies for advanced pharmacy practice in “patient care” [1].

Section Domain in the National Framework Competency
**Expert professional practice** Domain 7—Promote and contribute to optimal use of medicines Acquire expert knowledge and skills Use reasoning and judgement in assessing clinical situations Deliver accountable and flexible patient care Use professional autonomy
**Network, leadership and influence** Domain 2—Communication, collaboration and teamwork Use appropriate communication skills Engage in teamwork and consultation Work across boundaries Domain 3—Leadership and management Understand strategic context and contribute to strategic planning Understand and contribute to clinical governance Understand and contribute to the strategic vision Contribute to innovation and service development Motivate self and others Support and assist implementation of national priorities Understand and contribute to the effective use of resources Contribute to the identification and effective management of risk Promote improved performance Understand and undertake project management Understand change management principles and lead change Serve as a role model and mentor for others Domain 1—Professional and ethical practice Apply and monitor standards of practice Contribute to continuing professional development of self and others Domain 8—Critical analysis, research and education Conduct of education and training Links practice and education Educational policy Undertake critical evaluation activities Identify gaps in evidence base Design and deliver research projects to address gaps in the evidence base Apply research evidence into practice Supervise others undertaking research Establish research partnerships

Following the eight-week consultation period all feedback was collated, reviewed by the APPFSC in June 2012, and subsequently used to produce the final APPF. In August 2012 this was provided to the boards and councils of participating organisations for approval. All governing bodies of these pharmacy organisations subsequently approved the APPF. In an important initiative to secure the place of the APPF as a key resource for the future development of the profession, the APPFSC sought endorsement of the framework by the Pharmacy Board of Australia. Endorsement was granted in October 2012 and the published APPF was subsequently released to the profession.

In Table 4 the required competencies for advanced pharmacy practice in “patient care” are summarised. Note that in the APPF, each of the competencies are described at three levels—Transition level, Consolidation level and Advanced level. This is referred further in Section 4.2.

## 4. Discussion

### 4.1. Shaping the Framework Structure

The CoDEG Framework was noted to have arisen from a rigorous development process, is considered by its creators to be “a framework for clinical competence that will be generalisable across the profession” and is subject to ongoing evaluation [7]. Given its demonstrated flexibility for use by Australian pharmacists and proven adaptability to the Australian practice environment it was viewed as a suitable resource from which to develop an advanced practice framework for Australian pharmacists.

While it was noted that some health professions, for example nursing, had developed discrete sets of advanced level competencies, the APPFSC favoured adopting an approach that would demonstrate a continuum with the “general” level competencies since this was considered to:

(1) reinforce the concept that it is performance level that determines whether practice is advanced
(2) support the concept that acquired expertise and performance level operate in a continuum
(3) present advanced level competencies in a context that is more likely to promote professional development and growth in the pharmacy profession
(4) consolidate the value of the “general” level competency standards as a base for supporting professional practice and for facilitating progression towards achieving advanced practice.

The mapping work to compare the CoDEG Framework and the National Framework was useful to ascertain similarities in structure and content, and to confirm the continuum of competency standards between the “general” and “advanced” levels. The detailed mapping also supported the APPFSC’s view that the competency standards in the CoDEG Framework should be considered supplementary to those of the National Framework and further confirmed the usefulness of an adapted CoDEG Framework for describing and defining advanced practice in Australia.

### 4.2. Refinement of the Australian Advanced Level Framework

It is an accepted concept that newly registered pharmacists are at the beginning of their professional learning and that professional development is a career-long continuum of sustained learning and practice improvement. One of the noted strengths of the CoDEG Framework was its capacity to differentiate advanced pharmacy practice at three different performance levels in a continuum from “Foundation”, through “Excellence” to “Mastery”. Retention of this three-tiered structure in an Australian APPF was considered important for reinforcing the concept of a performance continuum associated with learning and career progression. It was also considered this would better support and guide the professional growth of pharmacists. However, a change in the terminology for the three levels was considered desirable to better reflect the intended progression of performance beyond “general” level. Initially, the levels were described as “Level A—Transition”, “Level B—Consolidation” and “Level C—Advanced”, however the prefix level descriptors A, B and C were removed in response to feedback received in the consultation phase with the profession.

Whilst noting that most advanced practice initiatives had occurred in patient care areas, the APPFSC was cognisant of the need to design a framework that would be sufficiently flexible to serve as a template for describing advanced practice expectations in all other areas of professional practice where it may be considered desirable to recognise advanced pharmacy practice. Therefore, the flexibility of the adapted CoDEG Framework was tested by using it to develop an APPF for “patient care” and then customising that Framework for the area of practice of “management and administration”. This process is further explained elsewhere [1] and not replicated here. This exercise demonstrated that the adapted framework could be used for any area of practice by changing or customising only those standards in the section labelled “Expert professional practice”. All other competency standards were considered to be universally applicable to advanced pharmacy practice regardless of the area of practice and customisation was therefore considered unnecessary. This perspective was supported by the finding that in the five frameworks developed, or under development, in Australia the most substantial content change had been made in the competencies of the “Expert professional practice” area. Ultimately, some minor changes in language in the competencies of other areas were deemed desirable to improve understanding.

### 4.3. Defining Scope of Practice and Prerequisite Competencies

The breadth of professional practice or “scope of practice” (as adapted from [8]) is defined as:

*A time sensitive, dynamic aspect of practice which indicates those professional activities that a pharmacist is educated, competent and authorised to perform and for which they are accountable*.

An exploration of the scope of practice of a pharmacist working in patient care at an “advanced” level and at a “general” level showed that the scope of practice for “advanced” level practice was significantly different to that which was generated for a pharmacist working at a “general” level, particularly in relation to Domain 3 “Leadership and management” and Domain 8 “Critical analysis, research and education” (refer to Table 1). The main differences were evident in those competencies, which are expected of more experienced pharmacists, rather than general level practitioners, such as leadership, organisational planning, personnel management, and high-level management of information systems and resources. A similar outcome was observed when the same exercise was conducted for the area of practice of “management and administration”. When comparing the two scopes of practice it became apparent that the competencies listed in the scope of practice for “general” level practitioner also defined the expected performance level of that practitioner. These competencies could, therefore, reasonably be considered to be the prerequisite competencies for progression to working with the “advanced” level competencies. This exercise demonstrated the usefulness of the National Framework for defining both the prerequisite “general” level competencies and the scope of practice of the “advanced” level practitioner and both were thought to be valuable components for inclusion in an APPF (outlined in Table 3). A full explanation (with accompanying tables) is available elsewhere [1] and has not been reproduced in this paper.

### 4.4. Describing General Career Expectations for Advanced Practitioners

In some of the five Australian advanced practice frameworks each of the performance levels had been further clarified by relating them to a pharmacist’s career progression or by providing statements of expectation in regard to issues such as experiential background, qualifications, level of clinical autonomy, peer recognition and influence on practice. This additional material was retained in the APPF. However, as the Framework was intended to be adapted and used in a wide range of practice areas, the APPFSC considered such information could only serve as a general guide. It has therefore been included in the Framework as non-mandatory “general characteristics of pharmacists working with advanced practice competency standards” to guide expectations and improve understanding of the likely experience and achievements at each of the three performance levels.

### 4.5. Finalising the APPF

The APPF was developed and published through the cooperative effort of the ten steering committee organisations. The APPFSC has proven to be a successful model for achieving outcomes on behalf of participating organisations and the pharmacy profession. The consultative approach adopted by the APPFSC generated the type of open discussion and honest feedback needed to improve the APPF. It also required that the members of the APPFSC move through some challenging discussions that are inevitably a part of this type of work with mutual trust and respect. The successful delivery of the APPF on behalf of all Steering Committee organisations and the profession is testimony to the extent to which this occurred and has been a rewarding achievement for this collaborative forum.

The APPF is “generic” in that it is intended to be used as a template for developing advanced practice frameworks for any area of professional practice where development and recognition of advanced pharmacy practice is considered desirable. To assist understanding of how the Framework works and how it can be adapted for use in specific areas of professional practice the published document contains a description of the component parts of the Framework together with two examples of its application to areas of practice, those being “patient care” and “management and administration”. Experience with its use will provide valuable insights as to whether the intended functionality has been achieved.

Customisation of the Framework for specific areas of advanced pharmacy practice primarily involves the addition of material to show the specific expert knowledge and skills required to meet the competencies in the “Expert professional practice” section of the Framework. The five frameworks reviewed during development of the APPF demonstrated that this material can be quite voluminous and detailed. In many instances it will be sufficiently detailed to serve as a “curriculum” for advanced practice training courses. Alternatively, parts of the material may be used to design appropriate and relevant continuing professional development activities. In both instances, customisation will have provided material that assists the ongoing development of the profession. In this regard, the Framework is a fundamental standards resource from which to move the profession forward. This is further demonstrated by the fact that it is suited for many of the same uses as the National Framework, including as an assisting resource for the development of job descriptions, evaluation of advanced practice training courses and recognition of advanced practice pharmacists.

Clearly, the APPF is also a fundamental standards resource for supporting the further development of individual pharmacists. The performance level of an individual is a function of the expertise of that individual which, in turn, is directly impacted by the learning and application to practice of new knowledge and skills. Changes in performance level occur along a continuum from “general” to “advanced” level. The linkage of the advanced practice competencies of the APPF to the “general” level competencies of the National Framework through using common Domain headers and the specification of prerequisite “general” level competencies from the National Framework is intended to show the profession that continuum. Individual pharmacists can therefore use the National Framework together with the APPF to review and assess their own performance, appreciate their existing capabilities, and identify opportunities for further professional development that will move them along the performance level towards advanced level practice. This is a pathway through which the profession more broadly can respond to the changing health care needs of the community.

### 4.6. Next Steps

The development of the APPF was a prerequisite for building the understanding and expertise needed to develop a model by which formal recognition of advanced pharmacy practice underpinned by the APPF could occur.

Work is currently in progress by the profession to develop a recognition model that is informed by other national and international initiatives to recognise advanced practice with the Australian Pharmacy Council acting as the independent assessment body for the credentialing of advanced practitioners.

A recognition model is the means by which advanced practice pharmacists can demonstrate to their peers and colleagues the achievement of advanced level practice. However, it is also the way the APPF can serve as a valuable tool for assuring members of the public of the competence of an advanced pharmacy practitioner and of the quality and safety of the professional services they provide. Thus, the application of the APPF in an advanced practice recognition model enhances the accountability of the profession to the public they serve.

## 5. Conclusions

The development of the APPF was heavily influenced by the CoDEG Framework and by those advanced practice frameworks in use, or under development, by Australian pharmacists. For those pharmacists who consider they are already working at an advanced level, or who have made significant contributions to earlier advanced practice framework initiatives, the development of an Australian APPF may seem long overdue. The APPFSC appreciates this perspective and acknowledges the exemplary work undertaken in developing advanced practice frameworks for specific areas of practice in Australia. However, it also recognises the importance of all pharmacy organisations and the profession moving forward together. Shared ownership of the achievement is demonstrated by the appearance of all logos of the participating organisations on the published APPF, and by its availability to all members of the profession through a neutral website established and maintained with funding from those organisations [9].

Although this paper reports specifically on an advanced practice initiative, which is relevant to Australian pharmacists, the Australian pharmacy profession learnt from the UK experience [10]. Our work may serve as an example or template for pharmacy professions in other countries. The resultant framework (APPF) is harmonised with both the Australian competency framework [2] and the CoDEG framework [4], emphasising the continuum between the general and advanced level competencies. As professional pharmacy practice continues to evolve it is of benefit for the profession to be able to learn from and inform each other.

## References

[1] Advanced Pharmacy Practice Framework Steering Committee (2012). An Advanced Pharmacy Practice Framework for Australia.

[2] (2010). National Competency Standards Framework for Pharmacists in Australia. http://www.psa.org.au/download/standards/competency-standards-complete.pdf.

[3] Royal Pharmaceutical Society of Great Britain (2009). Pharmacy practice 2008: Medicines focussed and patient centred. Pharmacy Practice Framework.

[4] Competency Development and Evaluation Group (2009). Advanced to Consultant Level Framework: A Developmental Framework for Pharmacists Progressing to Advanced Levels of Practice.

[5] Coombes I., Bates I., Duggan C., Galbraith K.J. (2011). Developing and recognising advanced practitioners in Australia: an opportunity for a maturing profession?. J. Pharm. Pract. Res..

[6] Council on Credentialing in Pharmacy (2010). Scope of contemporary pharmacy practice: Roles, responsibilities, and functions of pharmacists and pharmacy technicians. J. Am. Pharm. Assoc..

[7] McKenzie C., Borthwick M., Thacker M., Shulman R., Offord R., Tomlin M., Bates I., McRobbie D. (2011). Developing a process for credentialing advanced level practice in the pharmacy profession using a multi-source evaluation tool. Pharm. J..

[8] Queensland Nursing Council Scope of Practice—Framework for Nurses and Midwives (Amended June 2008). http://www.health.qld.gov.au/parrot/html/Documents/NursingScPrac.pdf.

[9] Advanced Pharmacy Practice. http://www.advancedpharmacypractice.com.au.

[10] Coombes I., Kirsa S.W., Dowling H.V., Galbraith K., Duggan C., Bates I. (2012). Advancing pharmacy practice in Australia: The importance of national and global partnerships. J. Pharm. Pract. Res..

